# Identification of candidate genes and clarification of the maintenance of the green pericarp of weedy rice grains

**DOI:** 10.3389/fpls.2022.930062

**Published:** 2022-07-22

**Authors:** Zhenyun Han, Fei Li, Weihua Qiao, Baoxuan Nong, Yunlian Cheng, Lifang Zhang, Jingfen Huang, Yanyan Wang, Danjing Lou, Jinyue Ge, Meng Xing, Weiya Fan, Yamin Nie, Wenlong Guo, Shizhuang Wang, Ziran Liu, Danting Li, Xiaoming Zheng, Qingwen Yang

**Affiliations:** ^1^National Key Facility for Crop Gene Resources and Genetic Improvement, Institute of Crop Sciences, Chinese Academy of Agricultural Sciences, Beijing, China; ^2^National Nanfan Research Institute (Sanya), Chinese Academy of Agricultural Sciences, Sanya, China; ^3^Guangxi Key Laboratory of Rice Genetics and Breeding, Rice Research Institute, Guangxi Academy of Agricultural Sciences, Nanning, China; ^4^International Rice Research Institute, Metro Manila, Philippines

**Keywords:** weedy rice, green pericarp, chlorophyll, BSA-seq, candidate genes identification

## Abstract

The weedy rice (*Oryza sativa* f. *spontanea*) pericarp has diverse colors (e.g., purple, red, light-red, and white). However, research on pericarp colors has focused on red and purple, but not green. Unlike many other common weedy rice resources, LM8 has a green pericarp at maturity. In this study, the coloration of the LM8 pericarp was evaluated at the cellular and genetic levels. First, an examination of their ultrastructure indicated that LM8 chloroplasts were normal regarding plastid development and they contained many plastoglobules from the early immature stage to maturity. Analyses of transcriptome profiles and differentially expressed genes revealed that most chlorophyll (Chl) degradation-related genes in LM8 were expressed at lower levels than Chl *a*/*b* cycle-related genes in mature pericarps, suggesting that the green LM8 pericarp was associated with inhibited Chl degradation in intact chloroplasts. Second, the F_2_ generation derived from a cross between LM8 (green pericarp) and SLG (white pericarp) had a pericarp color segregation ratio of 9:3:4 (green:brown:white). The bulked segregant analysis of the F_2_ populations resulted in the identification of 12 known genes in the chromosome 3 and 4 hotspot regions as candidate genes related to Chl metabolism in the rice pericarp. The RNA-seq and sqRT-PCR assays indicated that the expression of the Chl *a*/*b* cycle-related structural gene *DVR* (encoding divinyl reductase) was sharply up-regulated. Moreover, genes encoding magnesium-chelatase subunit D and the light-harvesting Chl *a*/*b*-binding protein were transcriptionally active in the fully ripened dry pericarp. Regarding the ethylene signal transduction pathway, the *CTR* (encoding an ethylene-responsive protein kinase) and *ERF* (encoding an ethylene-responsive factor) genes expression profiles were determined. The findings of this study highlight the regulatory roles of Chl biosynthesis- and degradation-related genes influencing Chl accumulation during the maturation of the LM8 pericarp.

## Introduction

**FIGURE 1:**
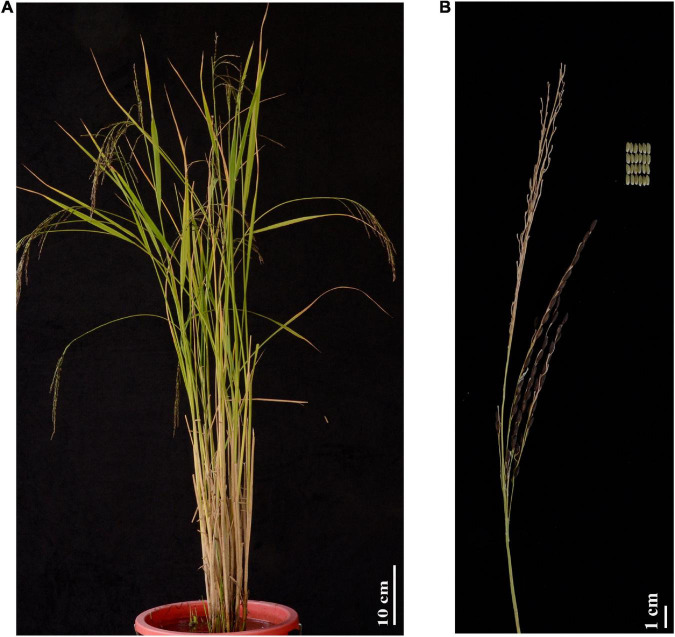
Phenotypic analysis of LM8. **(A)** Whole plant phenotype. **(B)** Panicle morphology and caryopsis color at the fully ripe stage.

During the Neolithic period, it took early farmers hundreds or thousands of years to transform wild plants into domesticated crops with increased yield and enhanced qualities (Buckler et al., 2001; Wing et al., 2018). However, domesticated plants and animals occasionally reacquire a portion of their wild-like traits during evolution (Ellstrand et al., 2010). This phenomenon, which is called de-domestication or feralization, has been observed in both livestock species and crops, including wheat (*Triticum aestivum*; Guo et al., 2020) and rice (Oryza sativa; Londo and Schaal, 2007; Qiu et al., 2017, 2020; Sun et al., 2019). Weedy rice (*Oryza sativa* f. *spontanea*), which is also called ‘red rice’, is a typical and convergent weed of cultivated rice. In addition to its weedy traits, weedy rice possesses additional novel mutations that are the result of natural selection. In our earlier studies, we collected rice germplasm and identified an unusual weed morphotype with a green pericarp and subsequently named it LM8. Besides the typical weedy characteristics such as hard and black hulls, seed shattering, persistent dormancy, shorter grain-filling stage and early maturity, etc., Because LM8 contains an AA genome and is closely related to cultivated rice (*japonica*-type; Li et al., 2021), it can be used to avoid any problems associated with distant hybridizations. Therefore, we selected LM8 as the starting material for further research into rice pericarp coloration. As shown in Figure 1, LM8 seeds are unique among rice germplasm resources because the pericarp is green from the early caryopsis development stage after flowering to the fully mature period, in which the panicle branch is withered and yellow. To further highlight the genetic characteristics underlying feralization that are beneficial for cultivated rice breeding, the phenotypic traits of LM8 were recently determined (Li et al., 2021). The distinct genetic basis of LM8 pericarp coloration is believed to have evolved during the de-domestication of cultivated rice varieties. Elucidating why the LM8 pericarp remains green will expand our understanding of the seed maturation processes of crops, while also potentially facilitating the breeding of rice varieties with novel grain colors. Therefore, there will likely be increasing interest in LM8 weedy rice in the future.

The pericarp color is an important agronomic trait and quality factor affected by domestication, with colored pericarps favored by consumers. The rice pericarp has evolved various colors, including red, purple, and black, which is the result of brown rice with a colored pericarp (Prathepha, 2009). Anthocyanins and proanthocyanins, which are the main pigments in black and red pericarps (Furukawa et al., 2006; Yang et al., 2019), have beneficial effects on human health and are synthesized in a branch of the flavonoid synthesis pathway (Saigo et al., 2020). Many studies on their biosynthesis pathway have revealed that the associated genes can be divided into two categories (i.e., structural and regulatory genes; Xia et al., 2021). One of the genes in the downstream part of the biosynthesis pathway encodes a dihydroflavonol reductase that catalyzes the conversion of colorless dihydroflavonols to leucoanthocyanidins (Furukawa et al., 2006). The subsequent reactions catalyzed by anthocyanidin synthase, leucoanthocyanidin dioxygenase, and UDP-flavonoid glucosyl transferase lead to the production of differentially colored anthocyanins. Cyanidin 3-*O*-glucoside is the most abundant pigment in purple or black rice (Furukawa et al., 2006; Zheng et al., 2019). The coloration of red rice is due to the accumulation of proanthocyanins, which are another subgroup of flavonoids. Proanthocyanin synthesis starts with reactions involving leucoanthocyanidin and cyanidin catalyzed by leucoanthocyanidin reductase and anthocyanidin reductase, respectively (Furukawa et al., 2006). The MBW complex comprises transcription factors from three families (MYB, bHLH, and WD40) that regulate different branches of the flavonoid biosynthesis pathway by recognizing promoter elements and activating the transcription of structural genes (Zheng et al., 2019). Rice includes a CAP regulation system, in which C, A, and P refer to a chromogen, activator, and tissue-specific regulator, respectively (Nagao, 1951; Xia et al., 2021). Additionally, C is encoded by a structural gene, whereas A and P are encoded by regulatory genes. Previous research indicated that *Rc* and *Pb/Ra*, which encode bHLH domain-containing transcription factors, are important for proanthocyanin and anthocyanin biosynthesis, respectively (Furukawa et al., 2006; Sweeney et al., 2006; Rahman et al., 2013). Furthermore, several genes encoding flavonone 3-hydroxylase, chalcone isomerase, and leucoanthocyanidin dioxygenase also influence rice pericarp coloration (Yang et al., 2019).

Changes in the color of ripening fruit are often associated with a concurrent degreening of the plant resulting from Chl degradation (Lai et al., 2015). Because Chl is also an important pigment in plants that undergo photosynthesis, Chl metabolism has been thoroughly investigated for more than 50 years. In contrast, the mechanism underlying the maintenance of green rice pericarps during the grain maturation process remains relatively unknown. Because of the increasing demand for crop production and the fact senescence usually affects crop yield and quality, there has been substantial research focused on leaf senescence in terms of chloroplasts and Chl metabolism, which has broadened our understanding of the green pericarp morphotype (Chen et al., 2013; Rong et al., 2013; Nguyen et al., 2020; Shin et al., 2020). For example, natural variations in the promoter of *Stay-Green* (*OsSGR*), which encodes a Chl-degrading Mg^2+^-dechelatase, are related to accelerated senescence and shortened lifespans in rice (Shin et al., 2020). Additionally, increases in Chl contents positively affect the rice biomass, photosynthetic rate, and grain yield (Wang et al., 2008). The expression of the ethylene-responsive genes *Sub1A* and *Sub1C* is reportedly up-regulated during Chl degradation in leaves (Fukao et al., 2006). The Chl degradation and biosynthesis pathways in rice leaves have been characterized (Sato et al., 2009; Liu et al., 2015; Nguyen et al., 2020). More specifically, Chl degradation is catalyzed by several catabolic enzymes. During the first step of Chl degradation, Chl *b* is converted to Chl *a* by non-yellow coloring 1 (NYC1) and NYC-like enzymes. Next, metal-chelating substance (MCS) and pheophytinase (PPH) remove Mg^++^ and phytol from Chl *a*, respectively. The resulting pheophorbide *a* is then converted to the primary fluorescent Chl catabolite by pheophorbide *a* oxygenase and red Chl catabolite reductase. Chloroplast development is closely linked with Chl metabolism in leaf cells. Almost 120 genes important for chloroplast development and function in rice have been identified and cloned.^1^ However, little is known about how the pericarp of fully mature rice grains remains green.

The LM8 morphotype provides researchers with a unique opportunity for elucidating the genetic basis of pericarp coloration and the extent to which it is the result of conserved or distinct mechanisms. To address the paucity of relevant information, in addition to LM8, a cultivar with a white pericarp (i.e., SLG) was analyzed in this study. Specifically, the chloroplast ultrastructure in LM8 and SLG pericarps was examined by transmission electron microscopy to investigate why the LM8 pericarp stays green. Additionally, the F_2_ population derived from a cross between LM8 and SLG was examined to identify the genetic factors responsible for the green pericarp. Moreover, a bulked segregant analysis (BSA-seq) and a transcriptome sequencing (RNA-seq) analysis were performed to preliminarily identify candidate genes underlying the green coloration of the pericarp. The study results indicate that the identified candidate genes responsible for the LM8 green pericarp trait may be useful for rice breeding and genetic analyses.

## Materials and methods

### Plant materials

Fresh pericarps were collected from LM8 (*Oryza sativa* f. *spontanea*) grains at 3, 6, 9, 12, 15, 18, and 21 days after full bloom (DAFB) for an examination of the chloroplast ultrastructure. At full maturity, LM8 and rice cultivar SLG (*Oryza sativa* L.subsp. indica), which has a white pericarp, were analyzed by transmission electron microscopy; the pericarps differed in terms of dryness. Rice grain maturation commonly involves the pre-milk, milk, dough, and yellow ripe stages. The LM8 grain developmental periods were divided into the following three stages: P1 (3, 6, 9, and 12 DAFB; fresh grain), P2 (15, 18, and 21 DAFB; fresh grain), and P3 (fully ripe; dry grain). The F_2_ population for the primary quantitative trait locus (QTL) analysis was generated following the hybridization between LM8 and SLG. It was grown in the experimental fields of Hainan under natural growth conditions in 2021. On the basis of the color and the Chl content of individual grain pericarps, the F_2_ population was divided into the following four subpopulations: dark green (C1), pale green (C2), brown (C3), and white (W). Twenty individuals were selected per subpopulation and combined to form a DNA pool.

#### Heat and light stress

After stripping the hull, fully ripened LM8 grains were retained for environmental stress experiments. A plant growth chamber was used to analyze the effects of different temperatures (25 and 40°C) and light conditions [24 h darkness and 24 h constant light (10,000–15,000 Lux white light from fluorescent tubes)]. The non-treated (control) and treated plant materials were immediately frozen in liquid nitrogen and stored at −80°C for the subsequent transcriptome analysis. The environmental stress experiments were performed using three biological replicates.

#### Determination of agronomic traits

Plant height and panicle length were measured using a ruler after sprouting. Three plants were randomly selected for measurements and then the average plant height and panicle length were calculated. The SC-G rice grain appearance quality image analysis system (Hangzhou WSeen Detection Technology Co., Ltd., Hangzhou, China) was used to determine the 1,000-grain weight, grain length, grain width, and grain length:width ratio.

#### Examination of chloroplast ultrastructure

Fresh pericarps were cut into small cubes, which were then immersed in alcohol acetate formalin mixed fixative at 4°C. The remaining sample preparation steps were completed as previously described by Li et al. (2014). The samples were examined using the H-7650 transmission electron microscope (Hitachi).

#### Measurement of chlorophyll content

After stripping the shell, 10–20 dry grains were ground, after which 0.8–1 g powdered material was treated with 80% (v/v) acetone under shade conditions. The Chl content was quantified using a published method (Jung et al., 2016). Three technical replicates were used to calculate the standard deviation.

#### DNA library construction and sequencing

Genomic DNA was extracted from young leaves according to the DNAsecure Plant Kit instructions. The concentration and quality of the two parental DNA samples and the four mixed DNA samples were determined using the Qubit fluorometer and by 1% agarose gel electrophoresis. The high-quality DNA samples were randomly fragmented via ultrasonic disruption. The generated DNA fragments underwent an end-repair step during which a poly-A tail was added to the 3′ end. After adding a sequencing linker, the modified fragments were purified and amplified by PCR. The constructed library was sequenced using the HiSeq Xten platform. The raw reads obtained by re-sequencing were aligned to the rice reference genome (Os-Nipponbare-Reference-IRGSP-1.0, MSU_v7.0) for the subsequent variation analysis.

#### Single nucleotide polymorphism detection and BSA-seq data analysis

The methods used for detecting single nucleotide polymorphism (SNPs) and analyzing sequences were previously described by Sun et al. (2018) and Zhao et al. (2021). Briefly, the Genome Analysis Toolkit (GATK; McKenna et al., 2010) was used for detecting SNPs. On the basis of where clean reads were aligned to the reference genome, the Picard toolkit was used for a de-duplication, whereas GATK was used for a local realignment and base recalibration to increase the quality of the SNP detection. The following SNP types were eliminated before the analysis: SNPs with multiple alleles, SNPs with a depth less than 4, SNPs with the same genotype in all mixed pools, and SNPs on recessive alleles that were not inherited from recessive parents. The genomic region harboring the mutation site and the effect of the mutation were determined according to the position of the variant site and the positional information for the genes in the reference genome.

The SNP-index was calculated to identify the candidate genomic regions associated with the production of a green pericarp. The SNP-index represents the proportion of reads harboring a SNP that differs from the reference sequence. The Δ(SNP-index) of each locus was calculated by subtracting the SNP-index of the non-colored pool from that of the colored pool. A SNP-index of 0 indicated all of the short reads contained genomic fragments from SLG, whereas a SNP-index of 1 indicated all of the short reads were from LM8. The average SNP-index was calculated for a 1-Mb interval according to a sliding window analysis with a 10-kb window. The statistical confidence intervals of Δ(SNP-index) were calculated under the null hypothesis of no QTLs as described by Takagi et al. (2013).

#### Gene mapping

The genomic region exceeding the threshold (90–99% confidence interval) was regarded as a candidate region for the target gene. Another criterion for selecting the candidate region was that it was repeatedly revealed by the results of different Δ(SNP-indices) associated with the same position. Furthermore, the selected candidate region was ideally annotated in the rice reference genome and related to plant Chl metabolism. To select the candidate gene as described by Yano et al. (2016), gene polymorphisms resulting in an amino acid change or altered splicing junctions and located in the 5′ or 3′ non-coding sequences flanking a coding sequence were preferred.

#### RNA library construction and sequencing

For the RNA-seq analysis, total RNA was extracted from the pericarp tissue using the RNAprep Pure Plant Plus Kit. The RNA samples were digested with the DNase I kit to eliminate any genomic DNA contaminants. The concentration and quality of each RNA sample were determined using the Qubit fluorometer and by 1% agarose gel electrophoresis. The RNA-seq analysis was performed using three biological replicates per sample. Each paired-end cDNA library was sequenced using the Illumina HiSeq 2500 system (50-bp single-end mode). After constructing the cDNA library, it was sequenced using the HiSeq Xten platform.

For the transcriptome analysis, clean reads were determined according to the annotated reference genome. Gene expression was analyzed using Bowtie2 (version 2.2.5), with expression levels calculated in terms of fragments per kilobase of exon model per million mapped reads (FPKM) using Cufflinks (version 2.1.1). Differentially expressed genes were detected using PossionDis (i.e., Poisson distribution) and the following criteria: fold-change ≥ 2.00 and false discovery rate ≤ 0.01. The differentially expressed genes were functionally characterized following Gene Ontology (GO) and Kyoto Encyclopedia of Genes and Genomes (KEGG) pathway enrichment analyses using MapMan (version 3.5.1). The default parameters of Expath (version 2.0) were used to assign GO terms to the differentially expressed genes. We considered only statistically significant (*p* < 0.05) GO terms for further analyses.

#### Semi-quantitative reverse transcription polymerase chain reaction

Total RNA was extracted from pericarps using the RNAprep Pure Plant Plus Kit and then treated with the DNase I kit to eliminate any genomic DNA contaminants. First-strand cDNA synthesized from the total RNA was used as the template for the sqRT-PCR analysis. The PCR conditions were as follows: 95°C for 5 min; 28 cycles of 94°C for 30 s, 57°C for 30 s, and 72°C for 30 s; 72°C for 7 min (final extension). The semi-quantitative reverse transcription polymerase chain reaction (sqRT-PCR) primers are listed in Supplementary Table 6. *Actin* (Lu et al., 2012) was used as an internal control to ensure an equal amount of cDNA was used for all sqRT-PCR amplifications.

## Results

### Non-degraded chlorophyll in intact chloroplasts was essential for the green pericarp

**FIGURE 2:**
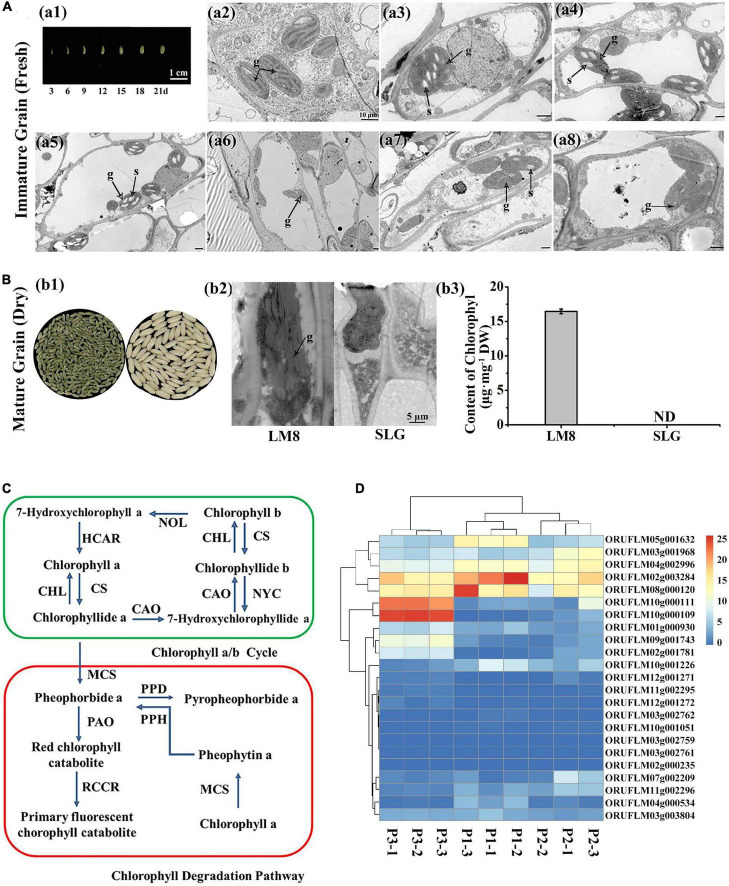
Transmission electron microscopy images and a heat map of chlorophyll *a*/*b* degradation-related gene expression during the maturation of the LM8 pericarp. **(A)** Transmission electron microscopy image of an LM8 fresh pericarp. **(a1)** Developmental changes in the pericarp appearance. Numbers represent the days after flowering. **(a2–a8)** Chloroplast ultrastructure in different caryopsis pericarp developmental periods; g, grana; s, starch. **(B)** Comparison of the chloroplast ultrastructure and chlorophyll content between the LM8 and SLG dry pericarps at the fully ripe stage. **(b1)** Morphological differences associated with pericarp colors. **(b2)** Chloroplast ultrastructure. **(b3)** Comparison of chlorophyll contents. **(C)** Proposed model for the chlorophyll cycle and chlorophyll degradation. **(D)** Chlorophyll *a*/*b* degradation-related gene expression heat map analysis. *MCS*: ORUFILM04g000534, ORUFILM09g001743; *PAO*: ORUFILM03g002761, ORUFILM03g002762, ORUFILM03g001968, ORUFILM02g001781, ORUFILM03g002759; *CAO*: ORUFILM10g000111, ORUFILM10g000109; *NYC*: ORUFILM02g003284, ORUFILM01g000930, ORUFILM03g003804; *PPD*: ORUFILM11g002295, ORUFILM12g001272, ORUFILM08g000120, ORUFILM07g002209, ORUFILM11g002296, ORUFILM12g001271; *RCCR*: ORUFILM10g001226; *CS*: ORUFILM02g000235, ORUFILM10g001051; *CHL*: ORUFILM05g001632; *HCAR*: ORUFILM04g002996.

To clarify why the LM8 pericarp is green, we examined the ultrastructure of chloroplasts in the mesophyll cells of fresh pericarps during the grain ripening period. There were no obvious differences between the LM8 and SLG chloroplasts, which were large and structurally normal at 3–21 DAFB, with distinct thylakoid membranes, stromal lamellae with small starch granules, and one or two plastoglobules (Figure 2A). These results suggested the green LM8 pericarp was the result of the accumulation of Chl in the complete chloroplast structure. At maturity, the difference in the pericarp color between LM8 and SLG (i.e., white pericarp) was most obvious (Figure 2B). To determine whether chloroplast morphology affects the pericarp color, we examined the chloroplast ultrastructure in pericarp mesophyll cells via transmission electron microscopy and noted any differences between LM8 and SLG. At maturity, all of the SLG chloroplast components were completely degraded, whereas the LM8 chloroplasts in the pericarp of dry grains contained indistinct thylakoid membranes as well as indistinct or no stromal lamellae (Figure 2B). Additionally, the Chl content of the LM8 pericarp was 16.45 μg mg^–1^, whereas Chl was not detected in the SLG pericarp (Figure 2B). Moreover, for LM8, there were significantly fewer thylakoid lamellae in the chloroplasts of mature and dry pericarps than in the chloroplasts of immature and fresh pericarps, but the structure of the thylakoid lamellae in the chloroplasts of mature and dry pericarps seemed normal and was similar to that of the thylakoid lamellae in the chloroplasts of immature and fresh pericarps (Figures 2A,B). Generally, the rice pericarp is often associated with the concurrent degradation of Chl, depending on whether a white or colored pericarp is produced. Hence, the transcriptomic profiles at three LM8 grain maturation stages were examined to analyze the expression levels of Chl degradation pathway-related genes (see text footnote 1) in the pericarp. A total of 26 genes related to Chl *a*/*b* degradation were identified on the basis of an analysis of enriched KEGG pathways (Figure 2C). Except for *PAO* (encoding pheophorbide *a* oxygenase), *PPH* (encoding pheophytinase), and *PPD* (encoding pheophorbidase), which had up-regulated expression levels, there were no obvious changes in the transcription profiles of the Chl *a*/*b* degradation-related genes in the LM8 pericarp (Figure 2D and Supplementary Figure 1). Both *NYC1* (encoding non-yellow coloring 1) and *CAO* (encoding chlorophyllide *a* oxygenase), which are involved in the Chl *a*/*b* cycle, had significantly increased transcript levels in mature LM8 grains. Thus, the green pericarp of LM8 may be mainly associated with a failure in the mechanism underlying Chl degradation in the intact chloroplast.

Specifically, a recent study demonstrated that the weedy rice pericarp appears red because of a lack of a 14-bp deletion in exon 6 of *Rc*, which leads to proanthocyanin biosynthesis and accumulation (Hamzah et al., 2020). A 2-bp (GT) deletion in the seventh exon of *Pb* on chromosome 4 is associated with the purple pericarp phenotype (Sakuraba et al., 2014). To confirm the main pigment in green pericarps is Chl, not proanthocyanins or anthocyanins, *Rc* and *Pb* allele polymorphisms were examined via a combined analysis of the available information regarding the LM8 and ‘Nipponbare’ genomes and phenotypic characteristics to determine whether LM8 can synthesize red or purple pigments. The LM8 *Rc* contains a 14-bp deletion in exon 6, which results in the production of a truncated protein lacking the bHLH domain that can turn the red pericarp white (Supplementary Table 2 and Supplementary Figure 2). Another *Pb/Ra* gene contains a 2-bp (GT) insertion in exon 7, leading to the conversion of a black pericarp to a white pericarp (Supplementary Table 2 and Supplementary Figure 2). These results reflect the lack of functional alleles for structural genes participating in anthocyanin and proanthocyanin synthesis. In other words, the green pericarp of LM8 appears to be unrelated to the production of red or purple pigments.

### Two complementary genes for the green pericarp were identified following a genetic analysis

**FIGURE 3:**
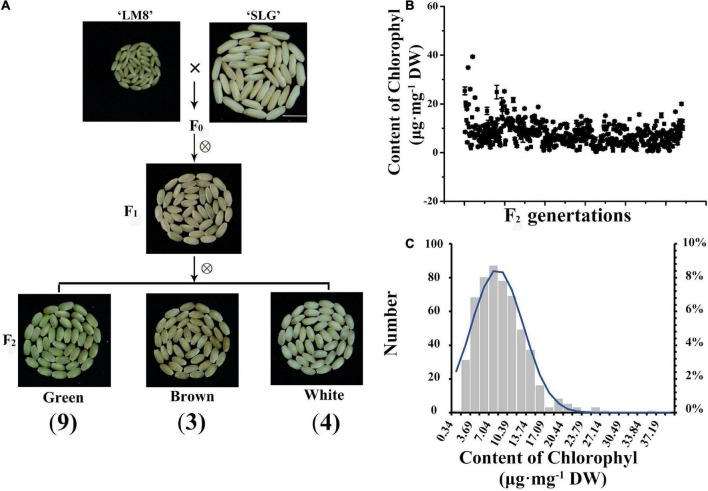
Segregation analyses of pericarp colors and chlorophyll contents of green pericarps in the F_2_ population. **(A)** LM8 (green pericarp) was crossed with SLG (white pericarp). The F_1_ grains produced green and brown pericarps. Additionally, F_2_ grains with green, brown, and white pericarps were detected. **(B)** Chlorophyll contents. **(C)** Normal distribution curve analysis.

To further clarify the genetic mechanism mediating the maintenance of a green LM8 pericarp, the F_0_ generation was self-pollinated, which generated F_1_ grains with white or green pericarps (Figure 3A). The selfing of F_1_ plants resulted in the production of F_2_ seeds with green, brown, or white pericarps (Figure 3A). To explore the genetic basis of the development of a green pericarp, the F_2_ population derived from the cross between LM8 and SLG was analyzed. The Chi-square (χ^2^) test was used to evaluate the goodness-of-fit of the number of genes segregating in the F_2_ generation. We hypothesized that the genes segregated according to the recessive epistasis ratio (9:3:4). First, among the 179 F_2_ progeny seeds in November 2020, the pericarp colors segregated in a 100:35:44 ratio (green:brown:white) (Table 1, Figure 3A, and Supplementary Figure 2). The results of the χ^2^ analysis of the F_2_ segregation data fit a 9:3:4 ratio (green:brown:white pericarps). With two degrees of freedom, the 5% critical value (5.991) was higher than the computed value (0.153). Hence, the gene segregation hypothesis (9:3:4) was not rejected (Table 1). The 542:267:299 ratio (green:brown:white pericarps) in May 2021 was consistent with the 9:3:4 ratio (Table 1, Figure 3A, and Supplementary Figure 3). According to the χ^2^ analysis, at the 95% confidence level, the *p-*value (5.991) was lower than the computed value (29.014) (Table 1). Thus, the gene segregation hypothesis (9:3:4) was rejected. Among the 682 F_2_ progeny seeds in November 2021, the pericarp colors segregated in a 486:177:257 ratio (green:brown:white) (Table 1, Figure 3A, and Supplementary Figure 3). At the 95% confidence level, the *p-*value (5.991) was higher than the computed value (5.098) (Table 1). Hence, the gene segregation hypothesis (9:3:4) was not rejected. The genetic segregation analysis confirmed that two major genes play a crucial role in Chl or chloroplast metabolism. The results were consistent with the findings of earlier research, suggesting that one critical gene (*Rc* or *Pb*) contributes substantially to anthocyanin and proanthocyanin biosynthesis (Furukawa et al., 2006; Sweeney et al., 2006; Rahman et al., 2013). Additionally, the intensity of the green coloration of the pericarp (light or dark) varied among 542 plants (Figure 3B). Subsequently, the accumulation of Chl was quantitatively analyzed (Figure 3B). Continuous variations were revealed by a normal distribution curve (Figure 3C). These results indicated two major genes and minor-effect genes likely control the production of a green pericarp in LM8.

**TABLE 1:** Chi-square (χ^2^) analysis of the segregation of pericarp colors in the F_2_ generation derived from a cross between LM8 and SLG.

Times	Number	Colored				χ^2^	
		Green	Brown	White	Total	χ^2^_(9:3:4)_	χ^2^_(0.05,2)_
2021.11	Observed	100	35	44	179	0.153	5.991
	Expected	101	33	45	179		
2022.5	Observed	542	267	299	1108	29.014	5.991
	Expected	623	208	277	1108		
2022.11	Observed	486	177	257	920	5.098	5.991
	Expected	517	173	230	920		

**FIGURE 4:**
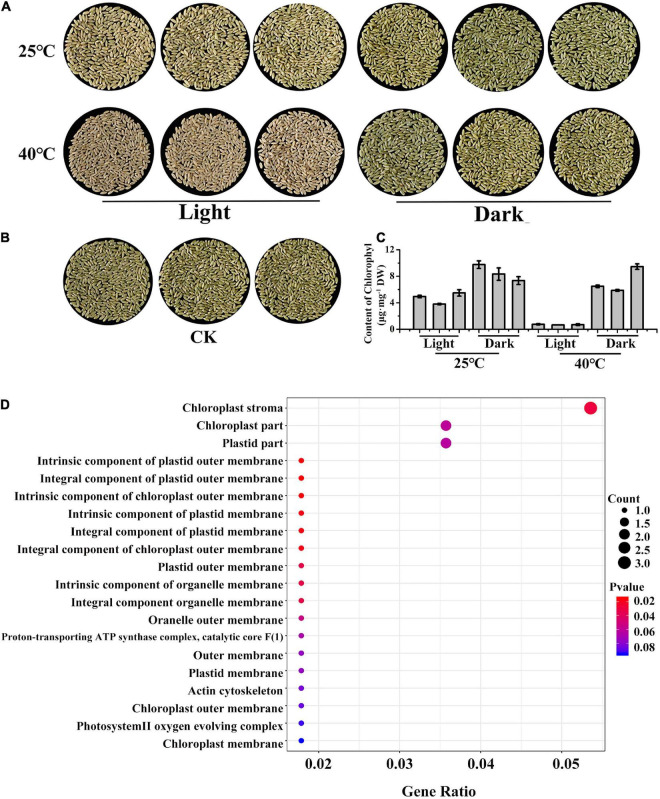
LM8 pericarp colors and chlorophyll contents in response to light and temperature stress. **(A,B)** Pericarp colors after a treatment with 24-h light and 25/40°C temperature stress. **(C)** Chlorophyll contents under stress conditions. **(D)** Dot plot analysis of enriched GO terms in the cellular component category following an RNA-seq analysis of samples that underwent the 24-h light and 40°C treatment.

Next, to determine why the gene segregation did not conform to the 9:3:4 ratio in May 2021, LM8 grains were exposed to light and heat stresses to analyze the effects of environmental factors on LM8 pericarp coloration at the transcriptome level. First, the Chl content was measured at 25/40°C under 24-h light and dark conditions. The brown pericarps (Figures 4A,B) and lowest Chl content (Figure 4C) detected at 40°C indicated that the high-temperature treatment accelerated Chl degradation under 24-h light, but not in darkness, resulting in the conversion of a green pericarp to a brown pericarp. These observations implied that under natural conditions, temperature is the dominant factor for maintaining green LM8 pericarps. Moreover, these results indicated that Chl contents are influenced by both light and temperature. A GO enrichment analysis of transcripts is an effective way to classify gene functions into three main categories (biological process, cellular component, and molecular function). Compared with the control, the genes assigned to the cellular component category that were responsive to a treatment at 40°C under 24-h light were enriched with the following GO terms: chloroplast stroma, chloroplast part, and chloroplast membrane (Figure 4D). Accordingly, the expression levels of 49 chloroplast-related genes (see text footnote 1) were examined at three maturation stages (P1, P2, and P3; Supplementary Figure 4). Compared with P1, the transcript abundance of chloroplast development-related genes (e.g., *TLP27*, *PIMT2*, *IM1*, and *trxm*) increased at full maturity, especially *INO1* and *hsp26* (Supplementary Figure 4). These findings were indicative of the importance of the chloroplast for the green pericarp of LM8.

### High-quality bulked segregant analysis sequences and mapped hotspot candidate regions

**FIGURE 5:**
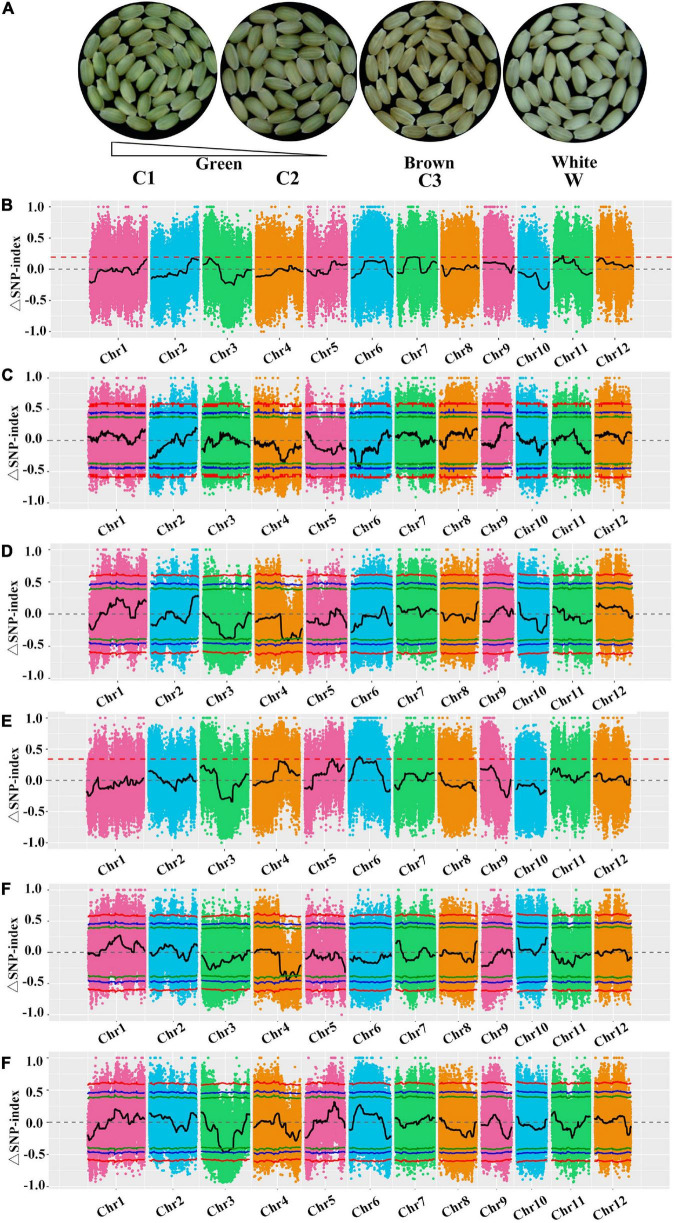
Bulked segregant analysis (BSA)-seq analysis for mapping genomic regions controlling green pericarps. **(A)** Comparison of the pericarp colors in four bulks. **(B)** Δ(SNP-index) plots between C1 and C2, **(C)** C1 and C3, **(D)** C1 and W, **(E)** C2 and C3, **(F)** C2 and W, and **(G)** C3 and W. The Y-axis presents the chromosomes, colored dots represent the calculated SNP-indices, and the black line is the fitted SNP-index. The green, blue, and red lines represent the 90, 95, and 99% threshold confidence intervals, respectively.

We used the BSA-seq method to detect major- and minor-effect genes. On the basis of the pericarp color and Chl content, the F_2_ population was divided into four subpopulations (Figure 5A), after which 20 lines per subpopulation were selected to generate the C1, C2, C3, and W DNA pools for the re-sequencing analysis. After filtering the re-sequencing data, 35,650,440 Mb (LM8), 42,627,732 Mb (SLG), 35,147,788 Mb (C1), 37,306,835 Mb (C2), 36,186,107 Mb (C3), and 34,327,052 Mb (W) clean data were retained (Supplementary Table 4). For these six samples, the Q30 was at least 93.15%, the average sequencing depth was 25×, and the lowest genome coverage was 93.63% (at one base coverage) (Supplementary Table 4). The BWA software was used to align the clean reads to the reference genome (MSU_v7.0), with alignment rates of 99.23, 98.03, 98.81, 99.02, 98.86, and 98.97% for LM8, SLG, C1, C2, C3, and W, respectively. These sequencing quality statistics reflected the diversity in the sequences and their relatively even chromosomal distribution, which were appropriate for the subsequent analysis.

A total of 7,345,087 SNPs were detected using GATK, including 301,680 in LM8, 1,347,589 in SLG, 1,430,628 in C1, 1,434,073 in C2, 1,431,100 in C3, and 1,400,017 in W. Many of the variant sites were upstream or downstream of genes or in the coding sequence region. After filtering, 1,002,151 high-quality SNPs were extracted, including 202,861 in C1–W, 202,769 in C2–W, 212,494 in C3–W, 187,970 in C1–C2, 196,057 in C1–C3, and 199,386 in C2–C3. Finally, on the basis of the SNP-index, 69 genome regions were identified (total length of 22 Mb) as significantly correlated with the green pericarp (Supplementary Tables 5, 6). The candidate regions were on chromosomes 3, 4, and 6, and comprised 3,403 genes with 1,596 non-synonymous SNPs (Supplementary Tables 5, 6). To eliminate false positive sites, the SNP-index for any two pools was analyzed according to the distance method. The distribution of SNP-indices in any two mixed pools and their Δ(SNP-indices) are presented in Figure 5. For the comparison of the candidate genomic regions between two pools using the 0.90 threshold, the intersecting regions were indicative of a significant association with the Chl/chloroplast (Figure 5 and Table 2). Ten candidate regions on chromosomes 3 and 4 included candidate genes related to the green pericarp (Table 2), of which *LOC_Os04g39970*, *LOC_Os04g39060*, *LOC_Os04g42030*, *LOC_Os03g29810*, *LOC_Os03g40020*, *LOC_Os03g40550*, and *LOC_Os03g38990* (i.e., seven known genes) were revealed to be associated with Chl accumulation and chloroplast degradation (Table 2).

**TABLE 2:** Information regarding the genomic hotspot region detected using the SNP-index algorithm.

Chromosome	Genomic candidate regions	Size (Mb)	Known genes	Candidate genes in LM8
Chr4	20820000–20870000	0.05		
Chr4	20920000–22650000	1.73		*ORUFILM04g002466* (*ERF110*), *ORUFILM04g002461* (*bHLH95*)
Chr4	22670000–22680000	0.01		
Chr4	22860000–23950000	1.09	*LOC_Os04g39970*, *LOC_Os04g39060*	
Chr4	24110000–26090000	1.98	LOC_Os04g42030	*ORUFILM04g001971* (*bHLH113*), *ORUFILM04g001857* (*DAD1*), *ORUFILM04g001996* (*DVR*)
Chr4	26270000–26290000	0.02		
Chr4	31600000–32800000	1.20		*ORUFILM04g001101* (*PIF4*), *ORUFILM04g001027*
Chr4	24180000–27310000	3.13	*LOC_Os04g42030*	*ORUFILM04g001864* (*MYB2*)
Chr4	30960000–32240000	1.28		*ORUFILM04g001159* (*CTR1*)
Chr3	15120000–23460000	8.34	*LOC_Os03g29810*, *LOC_Os03g40020*, *LOC_Os03g40550*, *LOC_Os03g38990*	*ORUFILM03g004308* (*CHL*), *ORUFILM03g004144* (*LHCB*)

### Identification of candidate genes related to the green pericarp

To identify candidate genes associated with the green pericarp, the expression patterns of the annotated genes in the LM8 pericarp were analyzed. More specifically, their expression levels in P1, P2, and P3, which corresponded to the milk, dough, and fully ripe grain stages, respectively, were determined. To more precisely identify candidate genes, the identified genes in SNP hotspot genome regions were screened and thoroughly annotated via a BLAST search of the non-redundant protein (NR), Swiss-Prot, GO, KEGG, and Clusters of Orthologous Groups of proteins (COG) databases.^2^ The most enriched GO terms in the biological process category were as follows: recognition of pollen (GO:0048544), response to stress (GO:0006950), regulation of programmed cell death (GO:0043067), regulation of cell death (GO:0010941), protein ubiquitination (GO:0016567), chloroplast organization (GO:0009658), protein localization to chloroplast (GO:0072598), and protein targeting to chloroplast (GO:0045036). The most enriched KEGG pathways were amino acid metabolism (ko00330) and purine metabolism (ko00230). Moreover, considering the green coloration of the pericarp is directly related to inhibited Chl/chloroplast degradation and increased Chl accumulation, the candidate genes related to the Chl/chloroplast were considered to potentially regulate the maintenance of a green pericarp.

**FIGURE 6:**
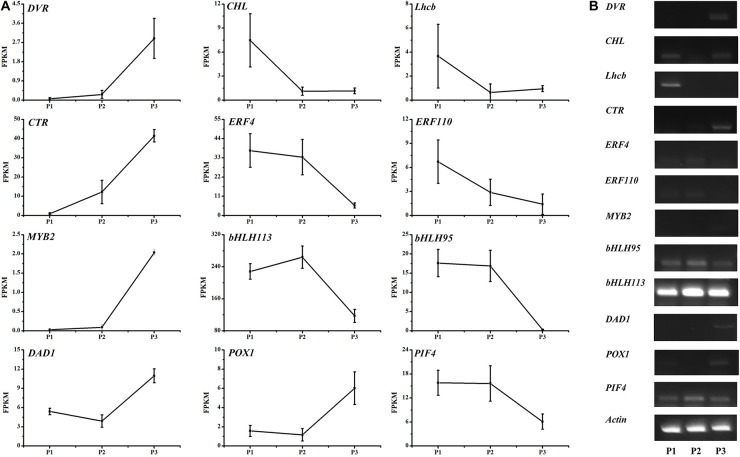
Expression levels of 12 differentially expressed genes in the LM8 pericarp during three developmental stages (P1, P2, and P3). Gene expression levels were analyzed on the basis of RNA-seq FPKM values and sqRT-PCR data. *Actin* was used as the control.

Three candidate genes encoding divinyl reductase (DVR), Mg chelatase I subunit D (CHLD), and light-harvesting complex II Chl *a*/*b*-binding protein (Lhcb II) were detected in the 15,120,000–23,460,000 bp region of chromosome 3 (Table 2). Both DVR and CHLD are key enzymes for Chl *a*/*b* synthesis. Moreover, DVR is the first key enzyme in the Chl cycle that converts divinyl chlorophyllide *a* to monovinyl chlorophyllide *a* (Figure 2) and then activates the cycling between Chl *a* and Chl *b*. Additionally, CHLD and Lhcb II are important for producing Chl *a*/*b* in the Chl synthesis pathway (Figure 2) and for harvesting light during photosynthesis, respectively. The expression of *DVR* increased significantly in the fully ripe period. The *CHLD* and *Lhcb II* transcription levels were slightly higher in P3 than in P2 (Figure 6).

On chromosome 4, the upstream transporter gene *CTR1* (negative regulator of the ethylene response pathway) and the downstream ethylene-responsive gene *ERF110* had the opposite expression patterns in developing LM8 grains (Figure 6). Notably, another ethylene-responsive gene, *ERF4* (an inhibitory transcription factor), was detected at a locus (30,939,170–30,940,275 bp) near the *CTR* region (30,960,000–32,240,000 bp). Moreover, the *ERF4* expression profile was similar to that of *ERF110* (Figure 6).

The MYB and bHLH transcription factors are involved in multiple biological processes, including flavonoid and anthocyanin pathways and responses to abiotic and biotic stresses. In this study, *bHLH113*, *bHLH95*, and *MYB2* were detected within the 21–26 Mb region on chromosome 4. In P3, *MYB2* expression increased significantly, whereas the expression levels of the two *bHLH* genes decreased sharply.

The *DAD1* gene encodes a protein that suppresses apoptosis. Its expression was previously confirmed to be triggered in host resistance-related responses (Sinha et al., 2015). However, in the current study, *DAD1* expression obviously increased in the senescent pericarp of fully ripened grains. The *POX1* gene encodes a peroxidase that functions with a catalase-peroxidase to produce guaiacyl lignin, syringyl lignin, and phenyl lignin in the phenylpropanoid biosynthesis pathway (Supplementary Figure 5). The *POX1* and *DAD1* expression levels were the same. Additionally, the *POX1* expression level was higher in brown pericarps incubated at 40°C under 24-h light than in the control brown pericarps. In maturing grains, the expression of *PIF4* (encoding phytochrome interacting factor 4), which was localized to a 1.2 Mb region (31,600,000–32,800,000 bp) on chromosome 4, was repressed. The encoded protein, which functions downstream of phytochrome B (i.e., light and temperature sensor), is an important part of a core signaling pathway that modulates the circadian clock and regulates plant growth.

## Discussion

Rice pericarp colors vary considerably (i.e., white, brown, red, purple, and black). In our study, the chloroplast ultrastructure in LM8 pericarp cells was examined using a transmission electron microscope (Figure 2). The results of this examination clarified why the LM8 pericarp remains green in fully ripened grains, while also providing insights into how Chl and chloroplast integrity is maintained. The KEGG analysis indicated that the expression levels of the genes in the Chl *a*/*b* degradation pathway were not down-regulated in LM8. Additionally, the RNA-seq and sqRT-PCR analyses revealed that the expression of Chl *a*/*b* cycle-related structural genes was up-regulated. We speculated that the observed expression of these genes influenced the accumulation of Chl in the green pericarp. In *Arabidopsis thaliana*, leaves that remain green at maturity are the result of *atsgr* mutations (Sakuraba et al., 2014) and the Golden 2-like (*SlGLK2*; Powell et al., 2012) transcription factor in tomato fruits, respectively. A previous study revealed that the development of a purple pericarp requires the expression of two genes, *Ra/PURPLE PERICARP* (*Pb*) and *PURPLE PERICARP A* (*Pp*), which contribute to anthocyanin accumulation, although purple pericarps subsequently turn white (Rahman et al., 2013). The *Pp* and *Pb* genes have complementary effects on the purple coloration of the pericarp, with a lack of *Pb* resulting in a brown pericarp (Rahman et al., 2013). Another study determined that *Kala1*, *Kala3*, and *Kala4* are also related to the production of purple pericarps (Oikawa et al., 2015). Functional alleles of *Rd* and *Rc* cooperatively mediate the development of red pericarps, whereas the expression of *Rc* alone leads to brown pericarps (Furukawa et al., 2006). The pericarps of F_2_ seeds were green, brown, white, green-brown, green-white, white-brown, and green-brown-white (Supplementary Figure 3). On the basis of these results, two complementary genes were presumed to be responsible for Chl synthesis in the green pericarp, but not the degradation of Chl or chloroplasts. One of the candidate genes (*DVR*) was previously isolated in rice mutant *824ys* and confirmed to be important for the production of monovinyl Chl *a* (Wang et al., 2010). Compared with the traditional method involving the fine-mapping and cloning of QTLs, high-throughput sequencing technology enables the efficient mapping of loci underlying the key agronomic traits of crops. In DongLanMoMi rice, several purple pericarp-related genes, such as *LOC_Os02g49140*, *LOC_Os12g07690*, and *LOC_Os06g17020*, were identified as candidate genes for anthocyanin biosynthesis following an analysis of F_2_ progeny using the new Pair-wise Comparison Analysis for Multiple Pool-seq (PCAMP) method (Yang et al., 2019). Additionally, the recently developed Rapid multi-QTL Mapping (RapMap) method, which involves the construction of a series of F_2_ gradient populations derived from multiple bi-parents with gradient phenotypes in diverse germplasms, was used to identify eight genes controlling the natural variations in rice grain length and width within 3 years (Zhang et al., 2021). The findings of the present study suggest two complementary genes regulate the green coloration of the pericarp of LM8 grains, which is in accordance with the results of earlier research. The 12 predicted genes identified by BSA-seq and RNA-seq analyses were considered as candidate genes on the basis of the transmission electron microscopy examination and transcriptome analysis. The functional characterization of these candidate genes may further clarify the development and maintenance of the green pericarp of rice grains.

In rice, the pericarp of the caryopsis is green immediately after flowering, but the subsequent decrease in Chl contents throughout the pericarp leads to color changes (e.g., red, purple, and black) or the elimination of color (white; Wang et al., 2020; Zhao et al., 2020). Chlorophyll is a highly photosensitive photosynthetic pigment, and temperature is also a critical abiotic factor influencing Chl accumulation (Ding et al., 2020). Previous investigations revealed that an exposure to heat stress induces a decrease in the Chl content of various plant species, including sorghum (*Sorghum bicolor*; Djanaguiraman et al., 2014), wheat (*T. aestivum*; Ristic et al., 2006), and Kentucky bluegrass (*Poa pratensis*; He and Huang, 2007). In fact, the effects of light and temperature on plant growth are related. Under natural conditions, plants are damaged by light and temperature conditions above or below an optimal range (Legris et al., 2017). Several studies on the light signaling pathway revealed key components that were responsive to heat stress (Franklin, 2009; Franklin et al., 2014; Lorenzo et al., 2016). In our study, a high temperature-mediated response induced by light resulted in Chl degradation, which explained the observed increase in the number of rice grains with brown or white pericarps in May 2021 (Supplementary Figure 3). In an earlier study on the brown pericarp of rice, Yang et al. (2018) revealed that serotonin biosynthesis is closely linked with the production of a dark brown endosperm in rice grains with high free lysine levels. Additionally, in the tryptophan metabolic pathway, tryptamine is converted to serotonin in the endosperm in a reaction catalyzed by T5H (Yang et al., 2018). Downstream of tryptophan, the pathway comprises two branches in the pericarp according to KEGG analyses, one involving the production of tryptamine and the other involving the production of serotonin (Supplementary Figure 6). Furthermore, *POX1* expression was up-regulated in brown pericarps during the 40°C and 24-h light treatment. Along with catalase-peroxidase, POX1 facilitates the production of guaiacyl lignin, syringyl lignin, and phenyl lignin (Supplementary Figure 5). Our data may be useful for future studies conducted to comprehensively characterize the mechanism underlying the brown pigmentation of the rice pericarp.

A de-domestication event enriches the evolution of crops and may lead to the production of important genomic resources relevant for breeding. Regarding weedy rice, the introduction of new mutations or the introgression of genetic variations from wild rice may have critical implications for species adaptations during de-domestication (Wu et al., 2021). Additionally, because of human-imposed directional selection, weedy rice has been subjected to diverse pressures. This has necessitated enhanced abiotic and biotic stress tolerance, including herbicide resistance (Merotto et al., 2016; Goulart et al., 2017), blast resistance, and cold or drought tolerance, as well as the ability to outcompete nearby crops for light and nutrients to ensure the survival of weedy rice in an agroecosystem. Moreover, a large biomass, compact and erect plant architecture, and lodging resistance are pre-requisites for high crop productivity (Figure 1). In addition to the genes underlying the green pericarp trait, we analyzed 13 genetic mutations associated with other traits (Li et al., 2017) in LM8 to explore their potential uses for developing novel rice varieties (Supplementary Table 2 and Supplementary Figure 2) and for clarifying the de-domestication evolutionary process. Three domestication genes were characterized on the basis of the domestication allele in LM8 (Supplementary Table 2), suggesting that LM8 was generated soon after de-domestication. Among the crop improvement-related genes, *An-1* (Luo et al., 2013) and *Bh4* (Zhu et al., 2011) were fixed or nearly fixed to the wild allele (Supplementary Table 2). In terms of the seed shattering trait of LM8, a SNP (T to G transversion) was detected in the region upstream of *qSH1*, suggesting that it affects seed shattering instead of *sh4* (Supplementary Table 2; Zhang et al., 2019). Moreover, LM8, which was identified in wild rice resources, has been exploited to improve agronomic traits, including quality and yield. Similar to the *SGR* gene, which controls the senescence and short lifespan of temperate *japonica* rice, the LM8 genome contains excellent alleles that may result in the development of traits conducive to yield increases (Supplementary Figure 2 and Supplementary Table 2). A relatively small grain may help clarify the mechanism regulating grain size from a different perspective. For example, six SNPs were identified in the first exon of *sh4*, whereas a long deleted fragment was detected upstream of *LG1* (Supplementary Figure 2; Zhu et al., 2013). Sequence alignment results revealed the substantial novel genetic variations in LM8, which may form the basis of future attempts to identify genes responsible for desirable traits (Supplementary Figure 2).

Compared with other weedy materials, LM8 is rich in mineral elements essential for humans (Yang et al., 2021). Moreover, chlorophyll (Chl) may help decrease the risk of developing colon cancer and breast cancer (Pierce et al., 2007; Frugé et al., 2019). Recent research indicated that Chl *a* derivatives, including sodium copper chlorophyllin, may have beneficial effects on human health when taken at therapeutic doses (Clark and Taylor-Robinson, 2021). Advances in molecular breeding technology and increases in health consciousness have resulted in a growing demand for crops with higher yield, better quality, increased stress resistance, and added health benefits. The pathways involved in pigment metabolism are increasingly being characterized, making it possible to breed novel rice varieties with desirable pericarp colors in an environmentally friendly manner to benefit humans’ health.

## Data availability statement

The RNA-seq data and genome resequencing data have been deposited at GenBank under BioProject ID PRJNA851542 and PRJNA851178, respectively. These dates can be found at: www.ncbi.nlm.nih.gov/bioproject.

## Author contributions

ZH and FL performed the research, analyzed the sequencing data, and wrote the first draft of the manuscript. QY, XZ, and DLi designed the study and edited the manuscript. WQ and BN supervised the project and provided experimental advice. YC, LZ, JH, YW, DLo, JG, MX, WF, YN, WG, SW, and ZL performed some of the seed germination experiments. All authors contributed and approved the final manuscript.

## Conflict of interest

The authors declare that the research was conducted in the absence of any commercial or financial relationships that could be construed as a potential conflict of interest.

## Publisher’s note

All claims expressed in this article are solely those of the authors and do not necessarily represent those of their affiliated organizations, or those of the publisher, the editors and the reviewers. Any product that may be evaluated in this article, or claim that may be made by its manufacturer, is not guaranteed or endorsed by the publisher.

## Footnotes

1
https://funricegenes.github.io/

2
http://blast.ncbi.nlm.nih.gov/Blast.cgi
