# Supplementary material for: Identification of candidate genes and clarification of the maintenance of the green pericarp of weedy rice grains
Source: Front Plant Sci. 2022 Jul 22;13:930062. doi: 10.3389/fpls.2022.930062 (PMC9354532; doi:10.3389/fpls.2022.930062)
Supplement: Supplementary file 1 [file Data_Sheet_1.docx]

Table S1 Phenotypic evaluation of traits for LM8

| Plant high (cm) | Flag leaf length (cm) | Flag leaf width (cm) | Panicle length (mm) | Thousand grain weight (g) | Grain length (mm) | Grain width (mm) |
| --- | --- | --- | --- | --- | --- | --- |
| 135.30±4.95 | 37.32±9.81 | 1.20±0.12 | 28.53±1.17 | 11.22 | 5.62±0.42 | 2.09±0.225 |

Table S2 The causation mutations of domestication and improvement genes in LM8

| Numbers | Gene name | Gene function | Mutation | | LM8 | | | Reference | |
| --- | --- | --- | --- | --- | --- | --- | --- | --- | --- |
|  |  |  | Wild type | Domestication or improvement allele | Mutation | Gene function | |  |  |
|  | **Domestication** |  |  |  |  | |  |  | |
| 1 | *sh4* | Seed shattering | G | T | T | | Shattering | | Zhang et al., 2019 |
| 2 | *LG1* | Closed panicle | G/C/C | A/T/T | A/T/T | | Closed | | Zhu et al., 2013 |
| 3 | *Prog 1* | Plant architecture | A/A | G/T | G/T | | Erect | | Jin et al., 2008 |
|  | **Improvement** |  |  |  |  | |  | |  |
| 4 | *Rc* | Pericarp color | WT | 14-bp deletion | 14-bp deletion | | Green pericarp | | Hamzah et al., 2020 |
| 5 | *C1* | Apiculus color | WT | 10-bp deletion | 10-bp deletion | | Black | | Qiao et al., 2021 |
| 6 | *An-1* | Awn length | WT | 1-bp deletion (most *indica* varieties) | WT | | No awn | | Luo et al., 2013 |
| 7 | *LABA1* | Awn barb | WT | 1-bp deletion | NF | | No awn | |  |
| 8 | *Bh4* | Hull color | WT | 22-bp deletion | WT | | Black | | Zhu et al., 2011 |
| 9 | *qSH1* | Seed shattering | G | T | G | | Shattering | | Konishi et al., 2006 |
|  | **Other selected** |  |  |  |  | |  | |  |
| 10 | *Rd* | Pericarp color | A-C mutation | | C | | Green pericarp | | Hamzah et al., 2020 |
| 11 | *Pb/Ra* | Pericarp color | GT insertion led to white pericarp | | GT insertion | | Green pericarp | | Rahman et al., 2013 |
| 12 | *PPKL1* | Grain length | C-A mutation at +1092 and C-T mutation at +1495 | | C and T | | Shorter | | Zhang et al., 2012 |
| 13 | *SGR* | Stay green | 8-bp insertion/deletion in promoter region | | 8-bp deletion | | Stay-green | | Shin et al., 2020 |

NF indicates Not Found in LM8 genome

Table S3 Summary of sequenced date analysis

| Samples | Total reads (Mb) | Clean reads (Mb) | Q30(%) | Depth (×) | 1×Coverage (%) | SNP number | Total Indel |
| --- | --- | --- | --- | --- | --- | --- | --- |
| C1 | 70,295,576 | 35,147,788 | 93.15 | 24 | 98.96 | 1,430,628 | 302,676 |
| C2 | 74,613,670 | 37,306,835 | 97.76 | 26 | 98.98 | 1,434,073 | 303,424 |
| C3 | 72,372,214 | 36,186,107 | 93.85 | 25 | 99.02 | 1,431,100 | 302,039 |
| W | 68,654,104 | 34,327,052 | 93.68 | 24 | 98.95 | 1,400,017 | 295,886 |
| LM8 | 71,300,880 | 35,650,440 | 95.11 | 24 | 97.47 | 301,680 | 73,877 |
| SLG | 85,255,464 | 42,627,732 | 95.08 | 28 | 93.63 | 1,347,589 | 283,721 |

Table S4 Summary of SNP-index algorithm date analysis

| Samples | Regions number | Size (Mb) | Gene number | Non-synonymous gene number | Confidence interval (%) |
| --- | --- | --- | --- | --- | --- |
| C1-W | 9 | 6.08 | 960 | 312 | 90 |
| C2-W | 2 | 4.41 | 702 | 168 | 90 |
| C3-W | 2 | 8.55 | 1260 | 832 | 90 |
| C1-C3 | 56 | 3.09 | 481 | 284 | 90 |
| Total | 69 | 22.13 | 3403 | 1596 |  |

Table S5 Information of the candidate region by SNP-index algorithm

| Bulks | Chromosome | Genomic candidate regions | Size (Mb) | Known genes | Candidate genes |
| --- | --- | --- | --- | --- | --- |
| C1-W | Chr4 | 20820000-20870000 | 0.05 |  |  |
|  | Chr4 | 20920000-22650000 | 1.73 |  | *LOC_Os04g34970*, *LOC_Os04g35010* |
|  | Chr4 | 22670000-22680000 | 0.01 |  |  |
|  | Chr4 | 22860000-23950000 | 1.09 | *LOC_Os04g39970*, *LOC_Os04g39060* |  |
|  | Chr4 | 24110000-26090000 | 1.98 | *LOC_Os04g42030* | *LOC_Os04g41570*, *LOC_Os04g43030* |
|  | Chr4 | 26270000-26290000 | 0.02 |  |  |
|  | Chr4 | 31600000-32800000 | 1.20 |  | *LOC_Os04g52770*, *LOC_Os04g53640* |
| C2-W | Chr4 | 24180000-27310000 | 3.13 | *LOC_Os04g42030* | *LOC_Os04g40930*, *LOC_Os04g42950* |
|  | Chr4 | 30960000-32240000 | 1.28 |  | *LOC_Os04g52140* |
| C3-W | Chr3 | 15120000-23460000 | 8.34 | *LOC_Os03g29810*, *LOC_Os03g40020, LOC_Os03g40550*, *LOC_Os03g38990* | *LOC_Os03g34230*, *LOC_Os03g36540, LOC_Os03g39610* |
|  | Chr3 | 23510000-23720000 | 0.21 |  |  |
| C1-C3 | Chr4 | 23016133-23043787 | 0.03 |  |  |
|  | Chr4 | 23048150-23048228 | 0.00 |  |  |
|  | Chr6 | 4272116-4276161 | 0.00 |  |  |
|  | Chr6 | 4282441-4494296 | 0.21 |  |  |
|  | Chr6 | 4498570-4519150 | 0.02 |  |  |
|  | Chr6 | 4519456-4526791 | 0.01 |  |  |
|  | Chr6 | 4534649-4534818 | 0.00 |  |  |
|  | Chr6 | 4535035-4537164 | 0.00 |  |  |
|  | Chr6 | 4540111-4662724 | 0.12 |  |  |
|  | Chr6 | 4663032-4664344 | 0.00 |  |  |
|  | Chr6 | 4684128-4687752 | 0.00 |  |  |
|  | Chr6 | 4712388-4712728 | 0.00 |  |  |
|  | Chr6 | 4712968-4715859 | 0.00 |  |  |
|  | Chr6 | 4716244-4759331 | 0.04 |  |  |
|  | Chr6 | 4794527-4798601 | 0.00 |  |  |
|  | Chr6 | 4801386-4809399 | 0.01 |  |  |
|  | Chr6 | 4835127-4864950 | 0.03 |  |  |
|  | Chr6 | 4869400-4873211 | 0.00 |  |  |
|  | Chr6 | 4904931-5126880 | 0.22 |  | *LOC_Os06g09810* |
|  | Chr6 | 5137620-5143383 | 0.01 |  |  |
|  | Chr6 | 5144563-5167331 | 0.02 |  |  |
|  | Chr6 | 5169089-6057135 | 0.89 |  | *LOC_Os06g10780* |
|  | Chr6 | 6059154-6060445 | 0.00 |  |  |
|  | Chr6 | 6060541-6088501 | 0.03 |  |  |
|  | Chr6 | 6089862-6096962 | 0.01 |  | *LOC_Os06g11500* |
|  | Chr6 | 6108592-6141466 | 0.03 |  |  |
|  | Chr6 | 6146325-6200685 | 0.05 |  |  |
|  | Chr6 | 6202451-6202801 | 0.00 |  |  |
|  | Chr6 | 6206381-6207152 | 0.00 |  |  |
|  | Chr6 | 6251956-6253942 | 0.00 |  |  |
|  | Chr6 | 6254765-6255876 | 0.01 |  |  |
|  | Chr6 | 6256661-6263045 | 0.00 |  |  |
|  | Chr6 | 6277733-6283395 | 0.01 |  |  |
|  | Chr6 | 6297656-6298596 | 0.00 |  |  |
|  | Chr6 | 6298802-6326780 | 0.03 |  |  |
|  | Chr6 | 6327033-6346141 | 0.02 |  |  |
|  | Chr6 | 6583426-6585264 | 0.00 |  |  |
|  | Chr6 | 6592279-7827549 | 1.24 | *LOC_Os06g13050* |  |
|  | Chr6 | 7836477-7838112 | 0.00 |  |  |

Table S6 Primers used for sqRT-PCR

| Genes number | Forward primer (5’-3’) | Reverse primer (5’-3’) |
| --- | --- | --- |
| ORUFILM08g000120 | CTCTCCCTCACCCCAGAAAACTATG | CTTCTTCTGTCTTAGCCTCTGTTGCC |
| ORUFILM12g001271 | CACTAGCTTCCATTCTACTCCGGC | CACAGCGACTTGAGGATGAGGTTG |
| ORUFILM12g001272 | TGGTTCAGGAGGGTTGAGTCTTATTC | CTGTTGGGATAATCTCTCTCTCTGG |
| ORUFILM11g002296 | CGAGTATGGAGATGTGTATGACCTGA | CCTCTTGGGGGCTTTGTTGATAG |
| ORUFILM11g002295 | GCTACGATGCTACCATTTGGACTTC | AGTGCTAATAGCTGTCACAGGCCAT |
| ORUFILM03g001968 | CGATGTTACCGAAGGAGTTTGAGG | CTGAATATCCCCATGCACCACTTG |
| ORUFILM01g000930 | GGAGTTGGATTTCAGTTATCAGGCG | GCTTTCCCTAATCCTCTTGTGCTTC |
| ORUFILM02g000235 | GAAAGATGAGGAGGAGGTGTTGGG | GAGGTTCCTCATGCACATGCAGTTG |
| ORUFILM10g001226 | CGATTGATTTCATGCTCCAGTCCTC | GAAGGTCCAGAAGCACCACGATC |
| ORUFILM04g002996 | GCTGATTTAGTGGTGGGCTATATGG | CTTCGGCCCAATCAGATTCAGTAG |
| ORUFILM05g001632 | GCTGGGCTAGGGATTGCTATTGTAA | GTATTGGAACTGAAAGACCACCTGAG |
| ORUFILM10g001051 | GTTCTTGCATGGATACCTCGCCTA | GTGGTATCCGGTCCAGACATCGTAT |
| ORUFILM02g003284 | GTTGGCAAAGGCAGTTGTGGAAAAG | CCCCAACCAGAGGACAAATTGACTA |
| ORUFILM09g001743 | GAGGTGGTGGCGGAGTGGAAGAAG | TGAAGCGTGGGAGGTTGGAGTGGAA |
| ORUFILM07g002209 | CTTCAGTGTCCATGAGACCTATCCC | GAGCTTGTGGCTTGGAGAAGAATG |
| ORUFILM10g000111 | CCAACTCCATATCTGCTTGCCCTCC | CATTTATCATCCGCTCTTGCTGCCC |
| ORUFILM10g000109 | CTGGTCCAGTGCAGCCATACAATCC | CTGAGCCAAGATGAAGAGGGCAAGC |
| ORUFILM03g003804 | GGATGATTGCATGTCGAGCATTCTG | TCTGGAGGTGACTCTTGATGTTGTG |
| ORUFILM02g001781 | CCTTCTCCTCTCTCCTCCTCCTCCC | CACTCCTCCCTCCAGTCGTATCTCGC |
| ORUFILM03g002762 | CAACATCCTGTGGTTCTACCCGAG | GGTCCATGAGATTCTCCACCAACAC |
| ORUFILM03g002761 | GAGCAGGGTGATATGGGTGTTTC | ATCTTTGGTAGGAGTTGTTGGCAGC |
| ORUFILM04g000534 | GCAATCACCAAAGGCACAAGAAG | TACATACATGTCAAGTGTCCGGTGG |
| ORUFILM03g002759 | GCTACGTGCCCACATCATCTGAC | CCTCCAGAGCTCTCATCCACTTC |
| *DVR* | CATCCTCCACCACGTCCCTGTTTG | ACGACAAAACACCCAATGTAGCCCG |
| *CHL* | CTCGGTTCATCCTCATTGGGTC | CCAAGGTTACTCCGAGCAGATG |
| *LHCB* | ATATGTGCAGGTATGGACCTGACCG | TTCTTGGACAGGATCTCCGGGAAGA |
| *CTR* | CAACGAGAGAAGGTTTGTAAGGGAG | GAGGATTTGACAGTATCGAGTTGCG |
| *ERF4* | CAGGTGCCCGCCTCGCCTTCGCT | CTACCTCCTTCTTGACGCTGACGAC |
| *ERF1110* | GTTCGTCCTCTGTTTCATGGCGGTA | GAAACCGTTGTATGCACTGTCACCG |
| *MYB2* | CGTGTTAGCTAGCTGGATCTATCGG | CTCTTCTAAACGCCTTCTCTACAGC |
| *bHLH95* | GTCGATGAAAGCCCATATCACACC | CACCTTCTCCTTCCTCTCTCTTGAC |
| *bHLH113* | GAGGTCGCCAATCACAGCAAAAG | CTGAAGAACAGAAGCAGTGTCCGTC |
| *PIF4* | CTGGATGAAGCGATCGACTACCT | GACGTTAGACTGTCTCTGTTGGGTG |
| *DAD1* | GACGGTGTTCCTATCTGATTCATTTCT | TCTTCATTCCATGTTGGTTCCTTCG |
| *POX1* | AAGGATCAGGTGGTTCTGCTAGGTG | TGTAGTAGCTGGTGTCGAACGTCCT |
| *Actin* | AGAGCTACGAGCTTCCTGATGGAC | GAGAGATGCCAAGATGGATCCTCC |

**
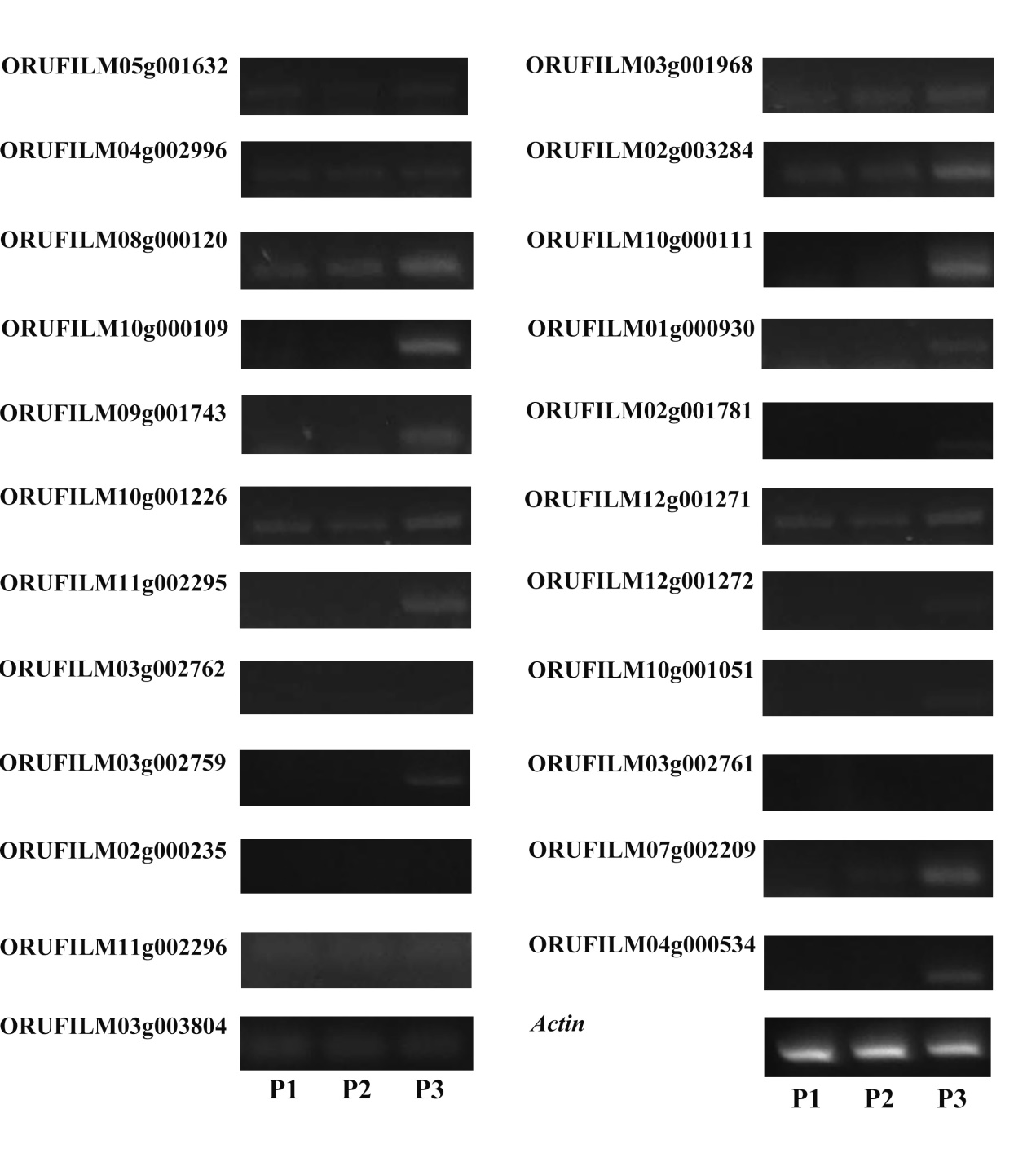
**

**Figure S**1 Results of the sqRT-PCR analysis of chlorophyll *a*/*b* degradation-related gene expression in the LM8 pericarp during three developmental stages (P1, P2, and P3). *Actin* was used as the control.


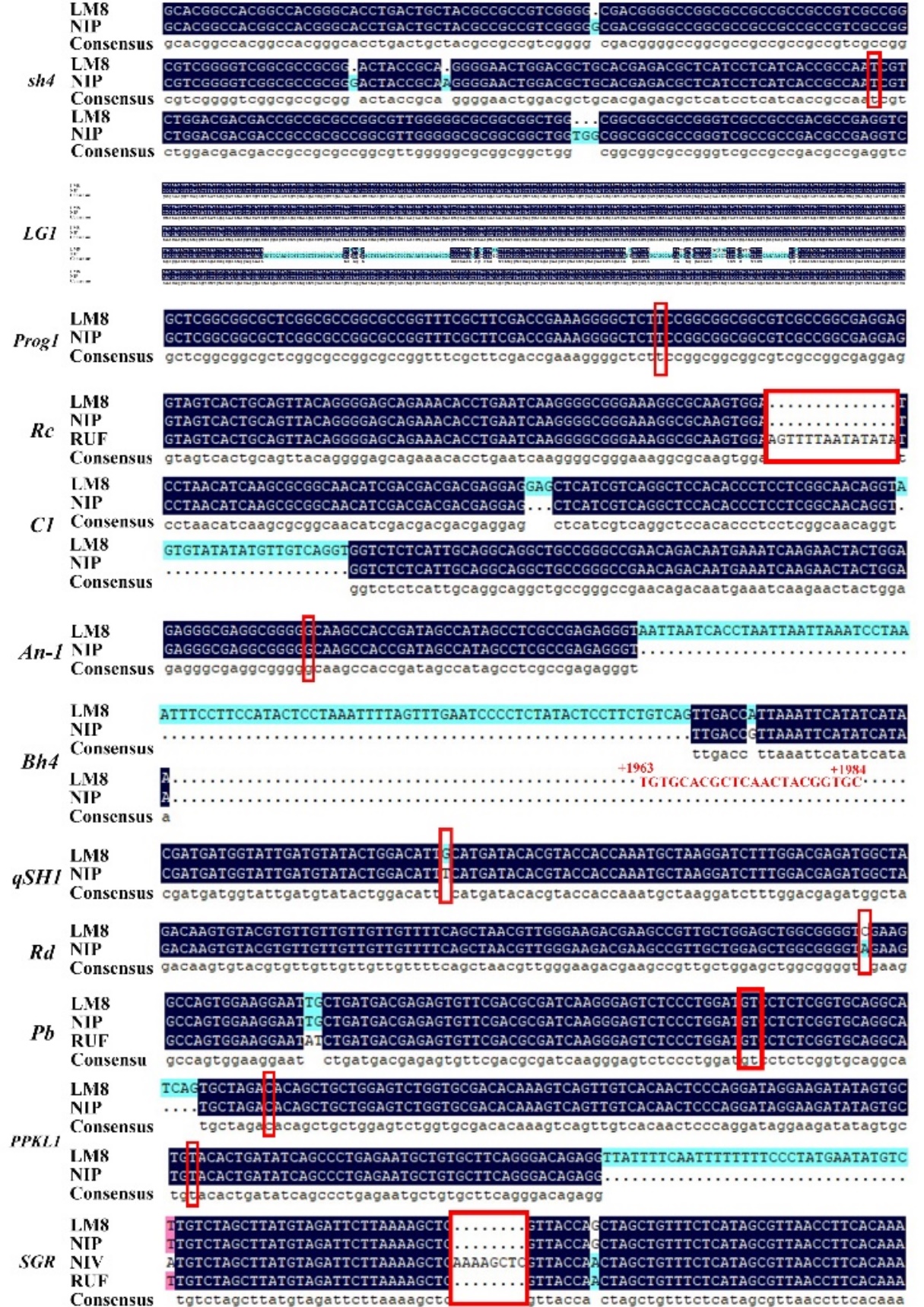


**Figure S2** Homologous gene sequence alignment. (A-M) Homologous gene sequence results for *sh4*, *LG1*, *Prog1*, *Rc*, *C1*, *An-1*, *Bh4*, *qSH1*, *Rd*, *Pb/Ra*, *PPKL1*, and *SGR*. NIP, Nipponbare (*Oryza sativa* spp. *japonica*); RUF, *Oryza rufipogon*; NIV, *Oryza nivara.* * indicates a termination codon. The mutation site is indicated by a red frame.

**
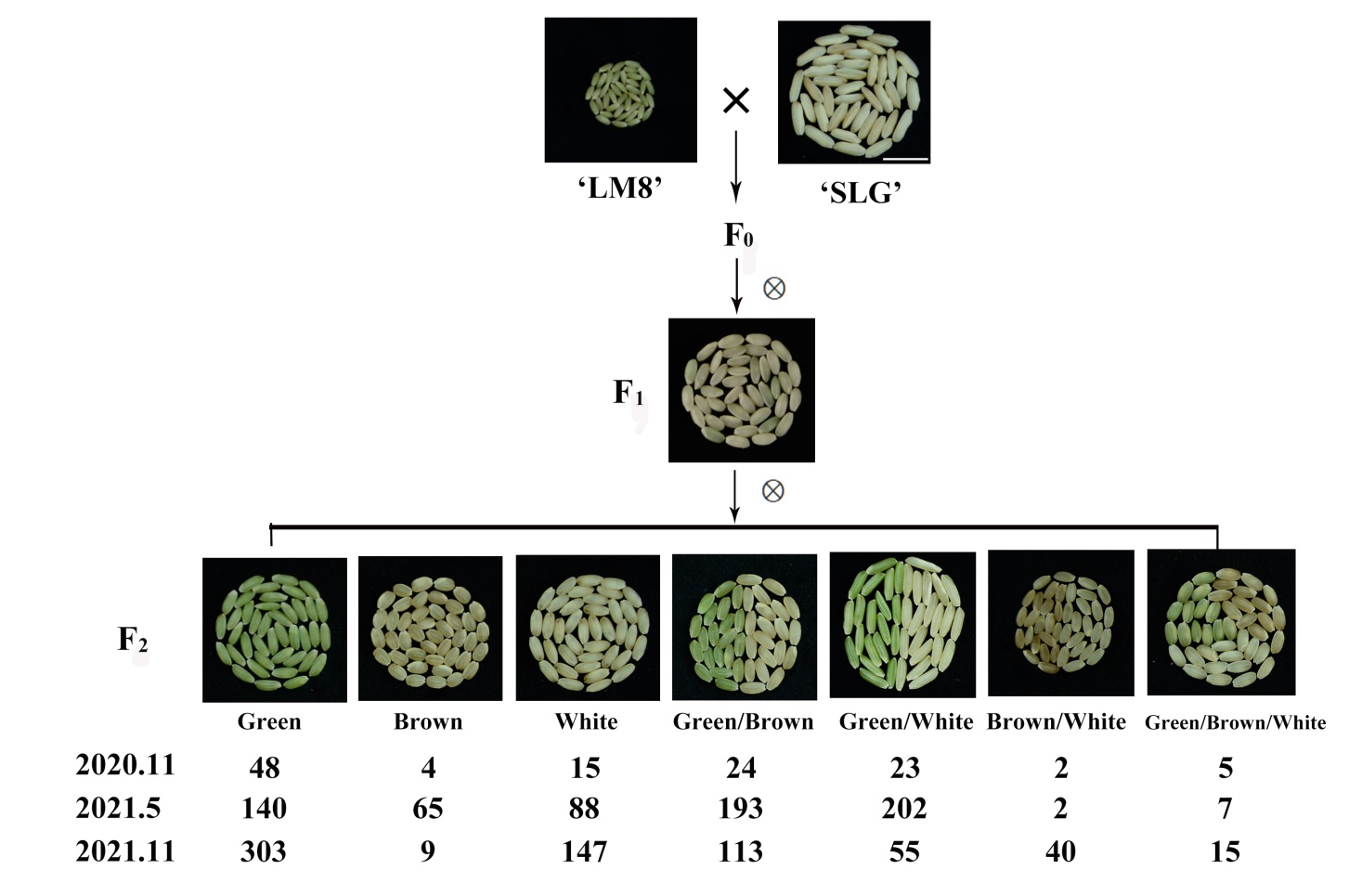
**

**Figure S3** Analysis of the segregation of pericarp colors. LM8 (green pericarp) was crossed with SLG (white pericarp). The number represents the number of plants in different years.


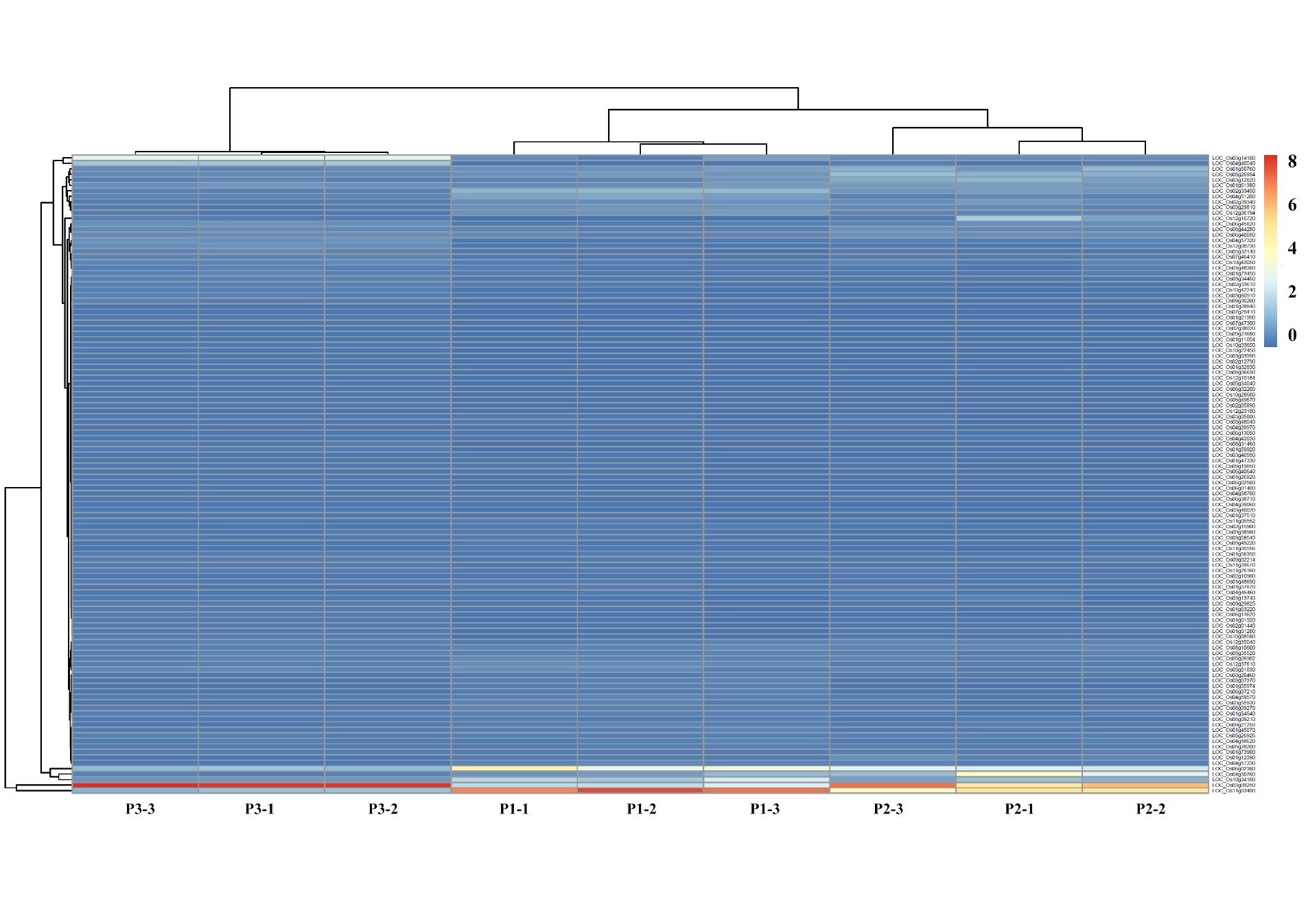


**Figure S4** Heat map analysis of chloroplast-related genes


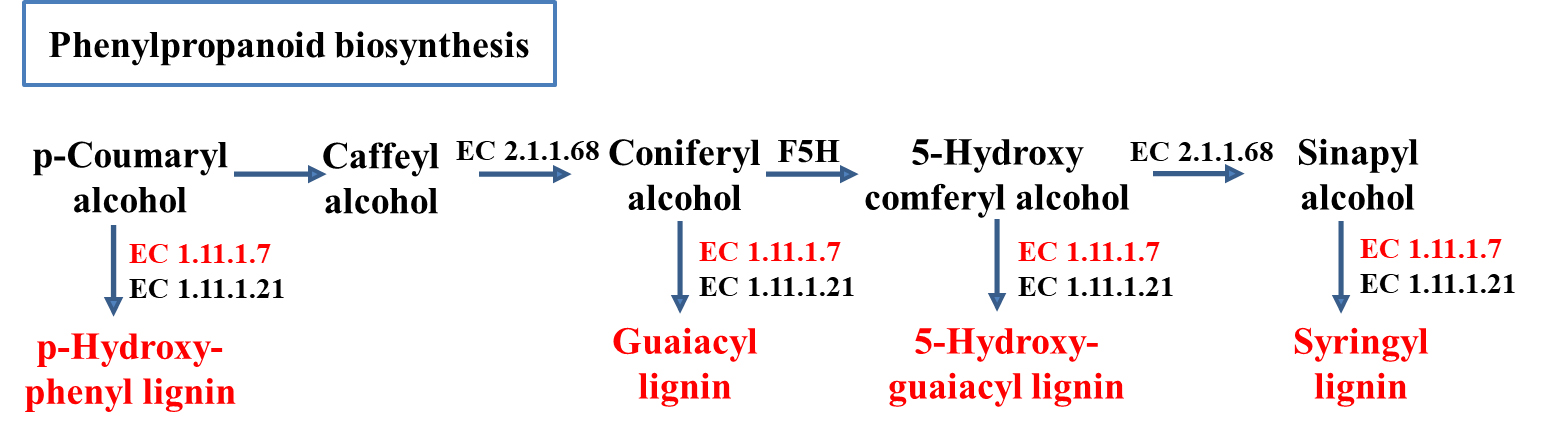


**Figure S5** Proposed model for tryptophan metabolism


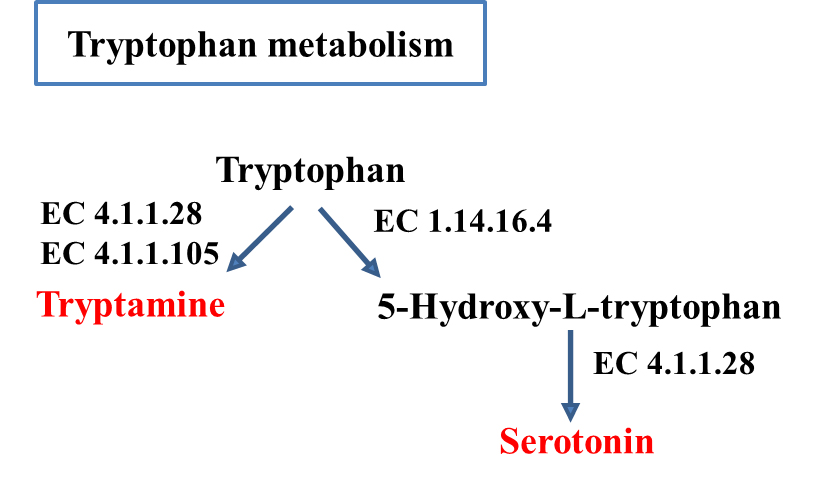


**Figure S6** Gene expression level analysis according to RNA-seq FPKM values.
